# Effect of puerarin in promoting fatty acid oxidation by increasing mitochondrial oxidative capacity and biogenesis in skeletal muscle in diabetic rats

**DOI:** 10.1038/s41387-017-0009-6

**Published:** 2018-06-12

**Authors:** Xiu-fang Chen, Lei Wang, Yong-zheng Wu, Shi-yu Song, Hai-yan Min, Yan Yang, Xuan He, Qiao Liang, Long Yi, Yong Wang, Qian Gao

**Affiliations:** 1 Center for Translational Medicine and Jiangsu Key Laboratory of Molecular Medicine, Medical School of Nanjing University, 210093 Nanjing, Jiangsu Province China; 2 Department of Biochemistry, School of Basic Medical Sciences, Wenzhou Medical University, 325035 Wenzhou, Zhejiang Province China; 3 Department of Anesthesiology, Affiliated Drum-Tower Hospital, Medical School of Nanjing University, 210008 Nanjing, Jiangsu Province China

## Abstract

**Background:**

Type 2 diabetes is characterized by dyslipidemia and the accumulation of lipids in non-adipose tissue, including skeletal muscle. Puerarin, which is a natural isoflavonoid isolated from the root of the plant *Pueraria lobata*, has been shown to have antidiabetic activity. However, the lipid-reducing effect of puerarin, in particular in skeletal muscle, has not yet been addressed.

**Methods:**

We examined the effect of puerarin on mitochondrial function and the oxidation of fatty acids in the skeletal muscle of high-fat diet/streptozotocin-induced diabetic rats.

**Results:**

Puerarin effectively alleviated dyslipidemia and decreased the accumulation of intramyocellular lipids by upregulating the expression of a range of genes involved in mitochondrial biogenesis, oxidative phosphorylation, the detoxification of reactive oxygen species, and the oxidation of fatty acids in the muscle of diabetic rats. Also, the effect of puerarin on mitochondrial biogenesis might partially involve the function of the μ-opioid receptor. In addition, puerarin decreased the trafficking of fatty acid translocase/CD36 to the plasma membrane to reduce the uptake of fatty acids by myocytes. In vitro studies confirmed that puerarin acted directly on muscle cells to promote the oxidation of fatty acids in insulin-resistant myotubes treated with palmitate.

**Conclusions:**

Puerarin improved the performance of mitochondria in muscle and promoted the oxidation of fatty acids, which thus prevented the accumulation of intramyocellular lipids in diabetic rats. Our findings will be beneficial both for elucidating the mechanism of the antidiabetic activity of puerarin and for promoting the therapeutic potential of puerarin in the treatment of diabetes.

## Introduction

Type 2 diabetes, which is categorized as a disorder of energy metabolism, is a disease of lipid metabolism, as well as a disease of glucose metabolism. Skeletal muscle, owing to its large mass (40% of body weight) and highly variable metabolic rates and demands on body energy supplies, makes a significant contribution to the homeostasis of glucose and fatty acid levels, which predominantly depends on the maintenance of effective mitochondrial function. Growing evidence suggests that mitochondrial dysfunction and the associated impairment of the oxidative capacity of skeletal muscle contribute to the development of insulin resistance^1–3^. Thus, the improvement of mitochondrial function in muscle is regarded as a potential therapeutic approach for the treatment of type 2 diabetes^4^.

Oxidative stress has been implicated in both the onset and the progression of diabetes and thus may be considered to indicate the extent of damage in patients. Diabetic subjects displayed an increase in the production of reactive oxygen species (ROS) and a decrease in the effectiveness of cellular antioxidant defense^5–7^. In the muscle of both diet-induced and streptozotocin (STZ)-induced diabetic mice, increases in the production of ROS were associated with mitochondrial dysfunction, and both the normalization of hyperglycemic status and antioxidant treatment that decreased the production of ROS restored the integrity of mitochondrial function^8^. Redox status may thus be an index of mitochondrial function.

Puerarin, which is a naturally occurring isoflavonoid, is abundant in the root of *Pueraria lobata* (Willd.) Ohwi, which has traditionally been used as a food and an herbal medicine in the treatment of diabetic conditions^9, 10^. Several studies have recently demonstrated that puerarin improved glucose intolerance^11^, decreased lipid contents in the serum and liver^12^ of high-fat diet (HFD) rodents, and reduced impairments in glucose and lipid metabolism in both *ob/ob*^13^ and STZ-induced diabetic mice^14^. However, the effects and mechanisms involved in the lipid-reducing function of puerarin in muscle have not yet been fully addressed. Here, we analyzed the effect of puerarin in terms of increases in oxidative capacity and biogenesis in mitochondria, which promoted the oxidation of fatty acids in skeletal muscle in HFD/STZ-induced diabetic rats.

## Materials and methods

### Materials

STZ, palmitate, puerarin powder (Cat. No. P5555), bovine serum albumin (BSA) (fatty acid-free), insulin (from bovine pancreas), and naloxone hydrochloride dihydrate were purchased from Sigma-Aldrich (St. Louis, MO, USA). Puerarin for injection (batch number 130902) was obtained from Baiyunshan Tianxin Pharmaceutical Co., Ltd. (Guangzhou, China). Antibodies against Shc (PG-797) were obtained from Santa Cruz Biotechnology (Santa Cruz, CA, USA). Antibodies against p-p66Shc (Ser36) (ab54578), SOD2 (ab68155), UCP2 (ab67241), PGC-1α (ab54481), CD36 (ab133625), SDHA (ab137040), ATP5A (ab14748), MTCO1 (ab14705), NDUFA9 (ab181381), NDUFS3 (ab177471), and UQCRFS1 (ab191079) were obtained from Abcam (Cambridge, MA, USA). Antibodies against SIRT1 (no. 9475), SIRT3 (no. 5490), ACC (no. 3662), p-ACC (Ser79) (no. 3661), AMPK-α (no. 5831), p-AMPKα (Thr172) (no. 2535), Akt (no. 9272), p-Akt (Ser473) (no. 9271), and β-actin (no. 4967) were purchased from Cell Signaling Technology (Beverly, MA, USA). Antibodies against CPT-1b (orb308786) were obtained from Biorbyt Ltd. (Cambridge, UK).

### Development of diabetic rat model and drug delivery

Six-week-old male rats (Sprague-Dawley) were obtained from the Zhejiang Center of Experimental Animals (Hangzhou, China). Rats were housed under conditions of controlled temperature (22 ± 2 °C) and humidity (50–60%) with an automatically controlled 12 h light–dark cycle and were provided with ad libitum access to rat chow and water. The experimental protocol was approved by the Laboratory Animal Administration Committee of Nanjing University and experiments were performed according to the university guidelines for animal experimentation. No sample size estimation or blinding was carried out in the experiments.

Rats were randomly allocated to groups with two dietary regimens, which were fed a low-fat diet containing 10% calories from fat (D12450J; Research Diets, Inc., New Brunswick, NJ, USA) or an HFD containing 60% calories from fat (D12492; Research Diets, Inc., New Brunswick, NJ, USA), respectively. After 5 weeks, the HFD rats were injected intraperitoneally (i.p.) with a single dose of STZ (35 mg kg^−1^), whereas the rats that received the low-fat diet (i.e., the control group) were given vehicle (citrate buffer, pH 4.4, i.p.). On the third day after being injected with STZ, rats with fasting blood glucose levels of ≥16.7 mmol l^−1^ were considered to be diabetic and were randomly subdivided into two groups, namely, a diabetic model group and a group treated with an injection of puerarin (100 mg kg^−1^, i.p.). The control rats and the diabetic model group were given the same volume of vehicle (5% propanediol, i.p.). The animals were treated daily for 4 consecutive weeks and were maintained on their respective diets throughout the treatment period. Body weights and food intake were recorded weekly and daily, respectively. All animal trials were repeated twice.

In order to observe whether μ-opioid receptor (MOR) was involved in the effect of puerarin on mitochondrial function, two further groups of diabetic rats were established using the abovementioned procedure. One group of rats were treated with puerarin (100 mg kg^−1^, i.p.), whereas the other group received an intramuscular injection of naloxone (10 μg kg^−1^) 30 min prior to treatment with puerarin (100 mg kg^−1^, i.p.). The animals were treated once a day for 4 weeks.

### Blood collection and tissue preparation

After puerarin had been administered for 4 weeks, fasted (12 h) rats were weighed and anesthetized (1% sodium pentobarbital, 30 mg kg^−1^, i.p.). Blood samples were collected from the vena cava. Serum samples were isolated after centrifugation of whole blood at 500×*g* at 4 °C for 15 min and were stored at −80 °C. The soleus muscle was promptly removed and divided into two sections, of which one was trimmed into strips with dimensions of 1 × 1 × 1 mm and fixed in 2.5% glutaraldehyde for analysis of the ultrastructure, and the other was immediately frozen in liquid nitrogen and stored in a freezer at −80 °C until use.

### Assays of blood metabolic indicators

Serum triglycerides (TG) and total cholesterol (TC) were measured by an automated biochemical analyzer (Olympus AU 600, Hamburg, Germany). Serum free fatty acid (FFA) levels, superoxide dismutase (SOD) activity, and malondialdehyde (MDA) levels were determined by colorimetric enzymatic analysis using commercially available kits (Nanjing Jiancheng Biological Corporation, Nanjing, China).

### L6 cell culture and treatments

Rat L6 skeletal muscle cells were purchased from the cell bank of the Chinese Academy of Sciences (Shanghai, China) and were grown in low-glucose Dulbecco’s modified Eagle’s medium (DMEM) supplemented with 1% penicillin/streptomycin (Gibco, Grand Island, NY, USA) and 10% fetal bovine serum (HyClone, Logan, UT, USA) in a 5% CO_2_ incubator at 37 °C. Confluent L6 cells were differentiated into myotubes by incubation with DMEM supplemented with 2% horse serum (HyClone) and were referred every 24 h. L6 myotubes were used for experiments after differentiation for 6–7 days.

Palmitate was prepared as previously described^15^ and was complexed with fatty acid-free BSA. To mimic hyperlipidemic conditions^16^, L6 myotubes were cultured in medium containing 0.75 mM palmitate for 24 h. Cells in the puerarin treatment group were pre-incubated with puerarin (0.3 mM) for 24 h followed by incubation with palmitate (0.75 mM) for 24 h. Control cells received 1.5% BSA (vehicle), as used in the palmitate treatment groups.

### Isolation of plasma membrane (PM)

PM preparations were obtained as previously described ^17^. In brief, muscle tissues or L6 myotubes were homogenized by passage through a 25 gauge needle in a hypotonic buffer (50 mM Tris–HCl, pH 8.0, 0.1% NP-40, 0.5 mM DTT, and protease and phosphatase inhibitors). After centrifugation at 1000×*g* for 10 min, the pellet was collected, washed twice with hypotonic buffer without NP-40, and then lysed in a similar hypotonic buffer containing 1% NP-40 for 1 h at 4 °C. The resuspended pellet was centrifuged at 16,000×*g* for 20 min. The supernatant was collected and used as the PM fraction.

### Real-time quantitative RT-PCR analysis

Total RNA from soleus muscle tissue or L6 myotubes was extracted with an RNeasy Mini Kit (Qiagen, Hilden, Germany) according to the manufacturer’s instructions and was quantified using a NanoDrop 2000 spectrophotometer (Thermo Fisher Scientific, Waltham, MA, USA). cDNA was synthesized with a PrimeScript^TM^ RT reagent kit (Takara, Osaka, Japan) using 200 ng RNA. Real-time PCR was performed using TaqMan^®^ Universal PCR Master Mix primers and probes (Roche, Shanghai, China) in a StepOne System (Applied Biosystems, Waltham, MA, USA). Calculations were performed by a comparative method (2^−ΔΔCT^) using β-actin as an internal control. The sequences of the primers used in this study are listed in Table 1.

**Table 1:** Primers used for the analysis of mRNA expression by real-time RT-PCR

Genes	Accession number	Primer sequence	Size (bp)
ACOX1	NM_017340.2	Forward: caccttcgagggagagaaca	75
		Reverse: cgcacctggtcgtagatttt	
LCAD	NM_012819.1	Forward: gcagttacttgggaagagcaa	77
		Reverse: ggcatgacaatatctgaatgga	
ACSL1	NM_012820.1	Forward: ttacacacgggggacattg	61
		Reverse: tcctgtcgataatcttcaaggtg	
CS	NM_130755.1	Forward: tttgcagcagctatgaatgg	78
		Reverse: ctgcgtcagccagacaag	
SIRT1	XM_006256146.2	Forward: ttcgtggagatatttttaatcaggt	111
		Reverse: ctggtaagttttcaccaaagaagac	
SIRT3	NM_001106313.2	Forward: tcccaggggaagacatacg	76
		Reverse: ttcacaacgccagtacagaca	
COIV	NM_017202.1	Forward: cactgcgcttgtgctgat	66
		Reverse: cgatcaaaggtatgagggatg	
TFAM	NM_031326.1	Forward: agctaaacacccagatgcaaa	111
		Reverse: tcagctttaaaatccgcttca	
MTCO1	YP_665631	Forward: tcatcccttgacattgtacttca	103
		Reverse: ggacgaagccagctatgatg	
UCP2	NM_019354.2	Forward: gactctgtaaagcagttctacaccaa	85
		Reverse: gggcacctgtggtgctac	
UCP3	NM_013167.2	Forward: gtaccgaagccccctacact	87
		Reverse: agaaaggagggcatgaatcc	
PGC1α	NM_031347.1	Forward: tgtggaactctctggaactgc	81
		Reverse: gccttgaaagggttatcttgg	
PPAR-δ	NM_013141.2	Forward: gaggacaaacccacggtaaa	104
		Reverse: catgactgacccccacttg	
β-actin	NM_031144.2	Forward: aaggccaaccgtgaaaagat	102
		Reverse: accagaggcatacagggaca	

### Western blot analysis

Protein was obtained from skeletal muscle tissue or L6 myotubes using radioimmunoprecipitation assay buffer containing a cocktail of protease and phosphatase inhibitors (Thermo Fisher Scientific). Protein concentrations were determined with a BCA Protein Assay Kit (Thermo Fisher Scientific). Equal amounts of protein (35 μg) from each sample were separated by 10% SDS-PAGE and were subsequently transferred onto polyvinylidene difluoride membranes (Millipore Corporation, Bedford, MA, USA). Antigens were visualized by sequential treatment with specific antibodies, secondary antibodies conjugated to horseradish peroxidase, and an enhanced chemiluminescence substrate kit (Millipore Corporation). The intensities of bands were quantified by densitometry using ImageJ software (NIH, Bethesda, MD, USA).

### Analysis of mitochondrial DNA (mtDNA)

Total DNA was extracted from soleus muscle tissue using a DNeasy Kit (Qiagen). The content of mtDNA was calculated using real-time quantitative PCR by measuring the threshold cycle ratio of a mitochondria-encoded gene (16S rRNA, forward 5′-cgattaaagtcctacgtgatctga-3′, reverse 5′-tgtcctttcgtactgggagaa-3′) vs. that of a nuclear-encoded gene (β-actin, forward 5′-aaggccaaccgtgaaaagat-3′, reverse 5′-accagaggcatacagggaca-3′).

### Analysis of muscle ultrastructure

Strips of soleus muscle or L6 myotubes were fixed as previously reported^8^. Ultrathin sections (thickness of 70–80 nm) were prepared, stained with uranyl acetate and lead citrate, and observed with a Hitachi-7500 transmission electron microscope.

### Statistical analyses

Data are expressed as the mean ± standard deviation (s.d.). Statistical analyses were conducted using SPSS for Windows version 14.0K (SPSS, Inc., Chicago, IL, USA). After confirming that all variables were normally distributed, the differences among multiple groups were analyzed by one-way analysis of variance, followed by a least significant differences multi-comparison test. Statistical differences between two groups were assessed using an unpaired two-tailed Student’s *t*-test. A *P*-value of less than 0.05 was considered to be statistically significant.

## Results

### Puerarin prevented body weight gain and alleviated hyperlipidemia in diabetic rats

**Fig. 1:**
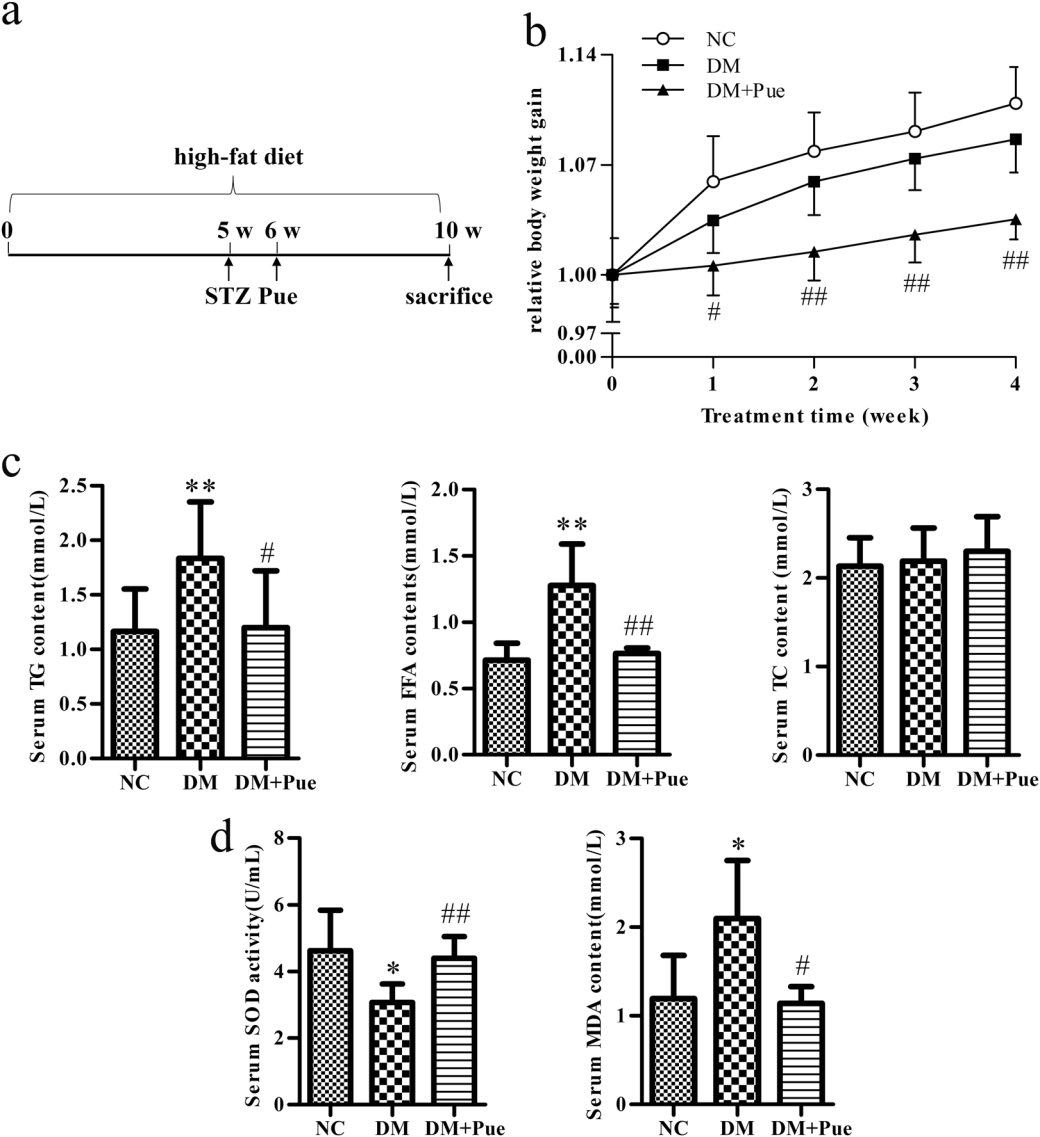
Puerarin prevented the body weight gain and alleviated hyperlipidemia in diabetic rats **a** A schematic representation of experimental process. **b** The body weight gain measurement. **c** Serum TG, FFA, and TC levels. **d** Serum SOD activity and MDA content. Data were presented as mean ± s.d., *n* = 8. ^*^P < 0.05, ^**^P < 0.01, vs. NC group; ^#^*P* < 0.05, ^##^*P* < 0.01 vs. DM group. *NC* normal control, *DM* diabetes mellitus, *Pue* puerarin

A rat model of HFD/STZ-induced diabetes^18, 19^ was initially established (Fig. 1a), and animals in this group displayed a similar trend in body weight gain to controls, whereas body weight gain was prevented to a significant extent after treatment with puerarin (Fig. 1b). There were no obvious differences in food intake between groups of diabetic rats with and without puerarin treatment (data not shown)^11, 12^, which might indicate an increase in energy consumption in animals treated with puerarin. Accordingly, serum levels of TG and FFA, but not TC, were significantly reduced in diabetic rats treated with puerarin. Otherwise, these levels were significantly increased in comparison with those in controls (Fig. 1c), which is a phenotype that closely reflects the typical energy metabolism disorders seen in type 2 diabetes^20, 21^. Correspondingly, a significant increase in serum SOD activity and a reduction in serum MDA levels were observed in diabetic rats treated with puerarin in comparison with diabetic rats without puerarin treatment (Fig. 1d). This indicated a beneficial effect of puerarin on both lipid metabolism and mitochondrial function.

### Puerarin decreased intracellular lipid accumulation in skeletal muscle

**Fig. 2:**
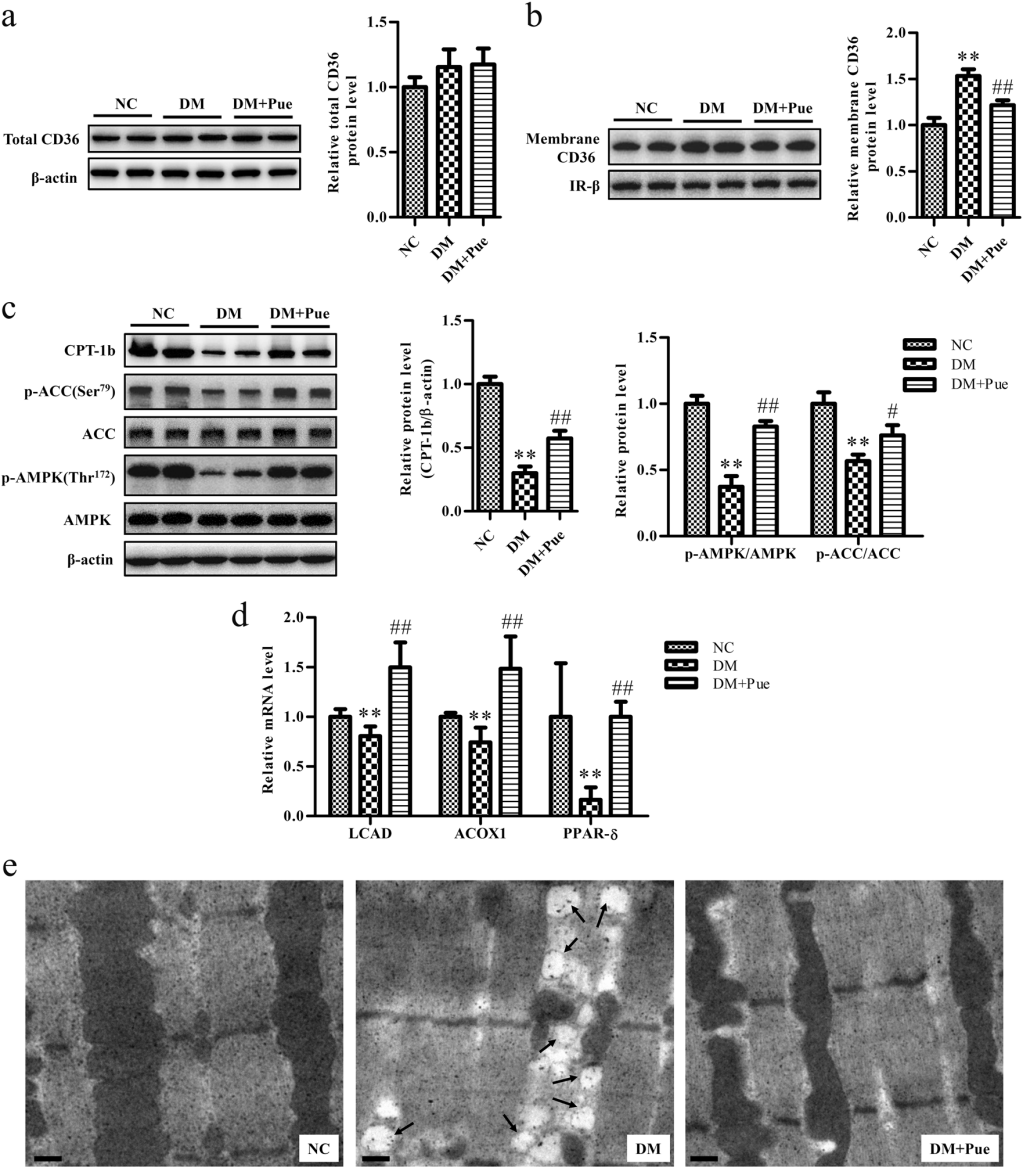
Puerarin decreased IMCLs accumulation in diabetic rats Representative blot and quantification of (**a**) total CD36 protein in muscle lysates, and (**b**) membrane CD36 in muscle sarcolemma. β subunit of insulin receptor (IR) was performed to confirm PM localization and normalization. **c** Western blot analyses and quantification of CPT-1b, p-AMPK (Thr172)/AMPK and p-ACC (Ser79)/ACC in muscle lysates. **d** Relative mRNA expressions of LCAD, ACOX1, PPAR-δ were analyzed by real-time RT-PCR and normalized to β-actin. **e** Representative TEM micrographs showed significant IMCLs accumulation (arrows) in diabetic rats and with much reduced IMCLs in puerarin-treated animals. Scale bar: 1 μm. Data were normalized to NC and presented as mean ± s.d., *n* = 5. ^**^P < 0.01, vs. NC group; ^#^*P* < 0.05, ^##^*P* < 0.01 vs. DM group

Because skeletal muscle accounts for a large proportion of body energy consumption, we next assessed the effect of puerarin on lipid metabolism in muscle. Firstly, levels of fatty acid translocase/CD36 were measured. Although there were no significant differences in total CD36 levels across the tested groups, membrane levels of CD36 were markedly increased in the muscle of diabetic rats in comparison with that of controls, which indicated increases in membrane translocation of CD36 and uptake of fatty acids (Fig. 2a and b), which was consistent with a previous study^22^. Secondly, we investigated the oxidation of fatty acids and showed that the expression of carnitine palmitoyltransferase 1b (CPT-1b) and levels of phosphorylation of AMP-activated protein kinase (AMPK) and acetyl-CoA carboxylase (ACC), as well as the mRNA levels of long-chain acyl-CoA dehydrogenase (LCAD), acyl-CoA oxidase 1 (ACOX1), and peroxisome proliferator-activated receptor-δ (PPAR-δ) were all significantly reduced in muscle in diabetic rats, accompanied by a significant accumulation of intramyocellular lipids (IMCLs) (Fig. 2c–e). Treatment with puerarin reversed these changes and led to a significant reduction in membrane CD36 levels and significant increases in the expression of the abovementioned genes involved in the oxidation of fatty acids (Fig. 2b–d). Importantly, puerarin significantly limited the accumulation of IMCLs in diabetic rats (Fig. 2e).

### The mitochondrial function of muscle was restored upon treatment with puerarin

**Fig. 3:**
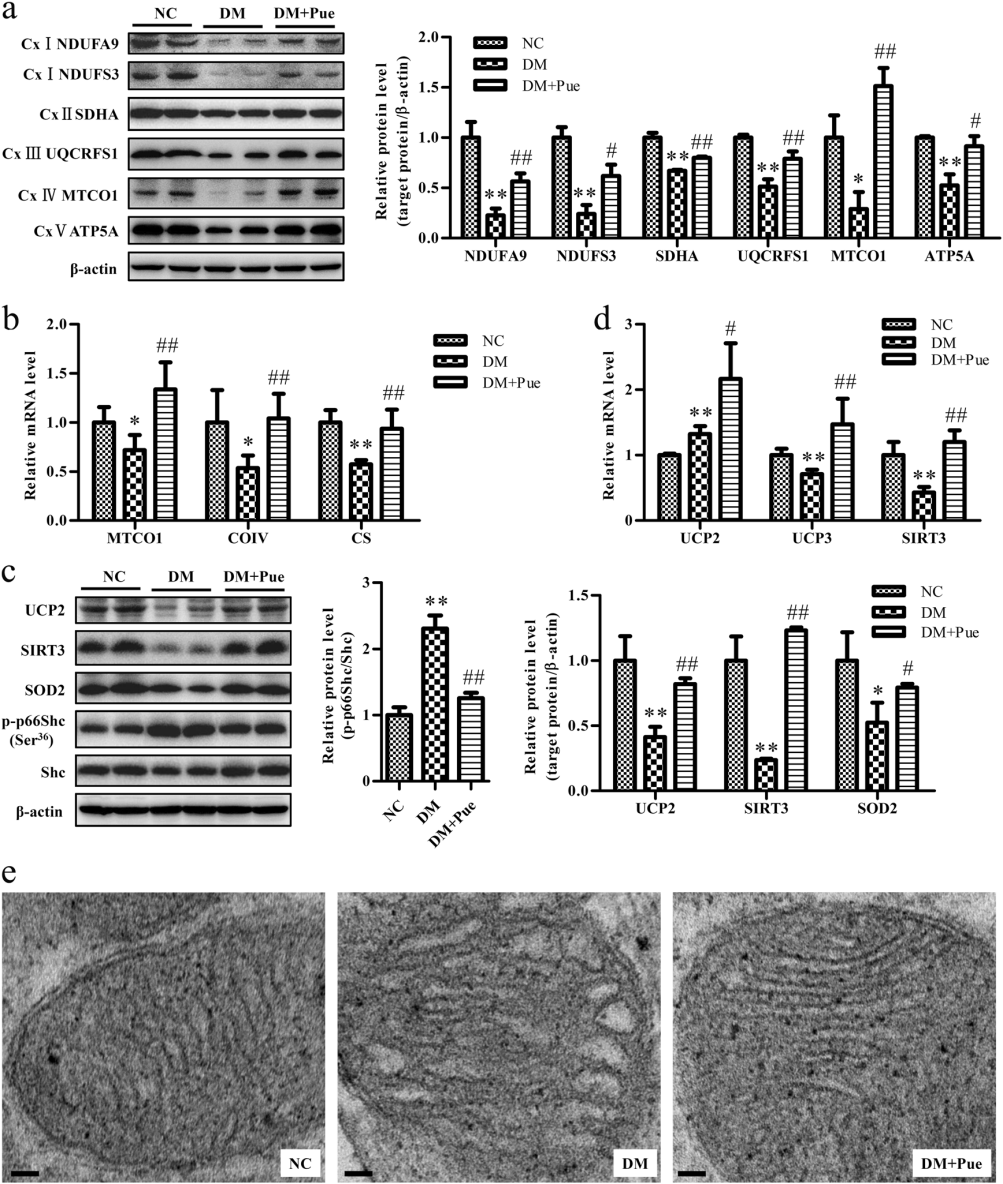
Puerarin restored mitochondrial function in the muscle of diabetic rats **a** Representative western blots and quantification of mitochondrial proteins used as markers for OXPHOS complexes (I–IV). **b** Real-time RT-PCR analysis of mRNA levels of mitochondrial proteins used as markers for electron transport chain (MTCO1, COIV) and TAC (CS) and normalized to β-actin. **c** Representative western blots and quantification of p-p66Shc(Ser36)/Shc, UCP2, SIRT3 and SOD2. **d** The mRNA levels of UCP2, UCP3, and SIRT3 were analyzed by real-time RT-PCR and normalized to β-actin. **e** Representative TEM images of mitochondrial ultrastructure from muscle. Scale bar: 0.2 μm. Data were normalized to NC and presented as mean ± s.d., *n* = 5. ^*^P < 0.05, ^**^P < 0.01, vs. NC group; ^#^*P* < 0.05, ^##^*P* < 0.01 vs. DM group

To examine the effect of puerarin on mitochondrial function in skeletal muscle, the expression levels of key components of the tricarboxylic acid cycle (TAC) and oxidative phosphorylation (OXPHOS) were measured. The expression levels of citrate synthase (CS; a key enzyme in the TAC) and subunits of mitochondrial complexes I–V, for example, NADH: ubiquinone oxidoreductase subunit A9 and core subunit S3 (NDUFA9 and NDUFS3, respectively; two subunits of complex I), succinate dehydrogenase complex flavoprotein subunit A (SDHA; a subunit of complex II), ubiquinol-cytochrome c reductase, Rieske iron–sulfur polypeptide 1 (UQCRFS1; a subunit of complex III), cytochrome c oxidase subunits 1 and 4 (MTCO1 and CO IV, respectively; two subunits of complex IV), and ATP synthase subunit alpha (ATP5A; a subunit of complex V) were all significantly decreased in the muscle of diabetic rats, whereas treatment with puerarin for 4 weeks restored the expression levels of these genes (Fig. 3a and b), which indicated an improvement in mitochondrial function.

Muscle in diabetic rats also exhibited higher oxidative stress, with an increase in the production of ROS and a reduction in the effectiveness of antioxidant defense, as evidenced by an increase in the phosphorylation of p66Shc and decreases in the expression levels of uncoupling proteins 2 and 3 (UCP2 and UCP3, respectively), sirtuin 3 (SIRT3), and SOD2. In contrast, these alterations were reversed in the muscle of diabetic rats treated with puerarin (Fig. 3c and d), which suggested an alleviation of mitochondrial oxidative stress. Moreover, muscle in diabetic rats exhibited abnormalities in mitochondrial ultrastructure, for example, swollen mitochondria with an increased number of disarrayed cristae and a reduction in electron density in the matrix, whereas puerarin restored the mitochondrial structure (Fig. 3e).

### Puerarin increased mitochondrial biogenesis in muscle involved in the function of MOR

**Fig. 4:**
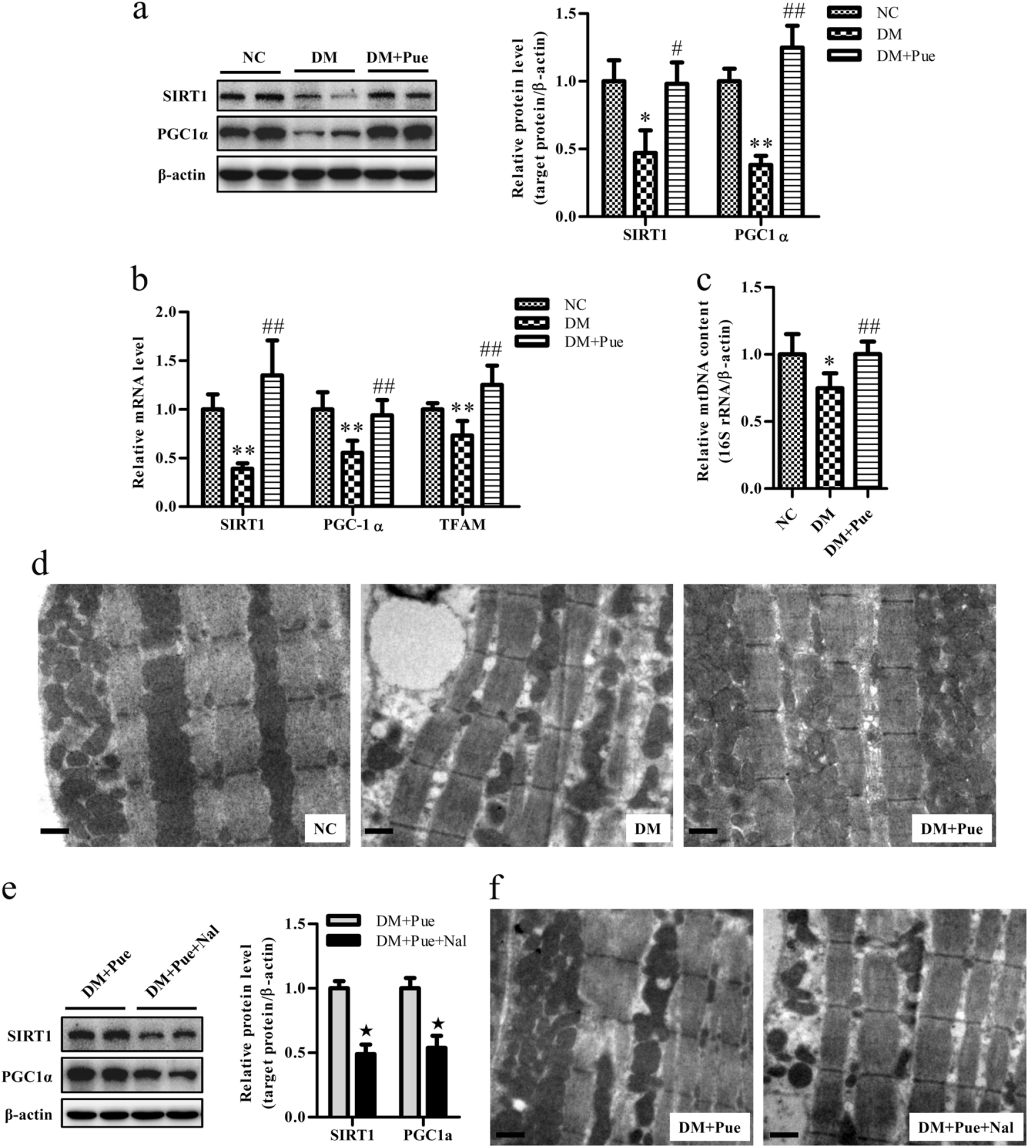
Puerarin increased mitochondrial biogenesis in muscle of diabetic rats **a**, **e** Representative blots and quantification of SIRT1 and PGC-1α. **b** Relative mRNA levels of SIRT1, PGC-1α, and TFAM analyzed by real-time RT-PCR and normalized to β-actin. **c** The mtDNA quantity evaluated as the ratios of 16S rRNA to β-actin determined by real-time PCR. **d**, **f** Mitochondrial density assessed by TEM in muscle of rats. Scale bar: 2 μm. Data were normalized to NC and presented as mean ± s.d., *n* = 5. (a–c) *P < 0.05, **P < 0.01, vs. NC group; ^#^*P* < 0.05, ^##^*P* < 0.01 vs. DM group. (e) ^★^ *P* < 0.01, vs. DM + Pue group. *Nal* naloxone

Impairments in mitochondrial biogenesis have been suggested to be a cause of reductions in the number and oxidative capacity of mitochondria in diabetes^23^. To assess the effects of puerarin on mitochondrial biogenesis in skeletal muscle, the expression of regulators of mitochondrial biogenesis, namely, sirtuin 1 (SIRT1), peroxisome proliferator-activated receptor gamma coactivator 1 alpha (PGC-1α), and mitochondrial transcription factor A (TFAM)^23, 24^, were analyzed. Significant decreases in the expression levels of these genes were observed in the muscle of diabetic rats, whereas treatment with puerarin restored the expression levels of these genes (Fig. 4a and b). Moreover, puerarin prevented reductions in the copy number of mtDNA to a significant extent, as reflected by the ratios of 16S rRNA to β-actin in the muscle of diabetic rats (Fig. 4c). Accordingly, transmission electron microscopy (TEM) showed that treatment with puerarin restored the number of mitochondria in the muscle of diabetic rats, which otherwise underwent a significant decrease (Fig. 4d).

Previously, Chen showed that the glucose-reducing effect of puerarin in rodents was dependent on MOR^25^. In addition, we found that puerarin could act on skeletal muscle to improve insulin signaling in diabetic rats involving the action of MOR^17^. To determine whether MOR is also involved in the effect of puerarin on mitochondrial biogenesis, the MOR antagonist naloxone was used. As was expected, the enhancements in the expression levels of the regulators of mitochondrial biogenesis SIRT1 and PGC-1α (Fig. 4e) and the increase in the density of mitochondria (Fig. 4f) induced by puerarin in the muscle of diabetic rats were significantly reduced by pretreatment with naloxone, which indicated the involvement of MOR signaling in the effect of puerarin on mitochondria.

### *In vitro* studies revealed a direct effect of puerarin on the oxidation of fatty acids in myotubes

**Fig. 5:**
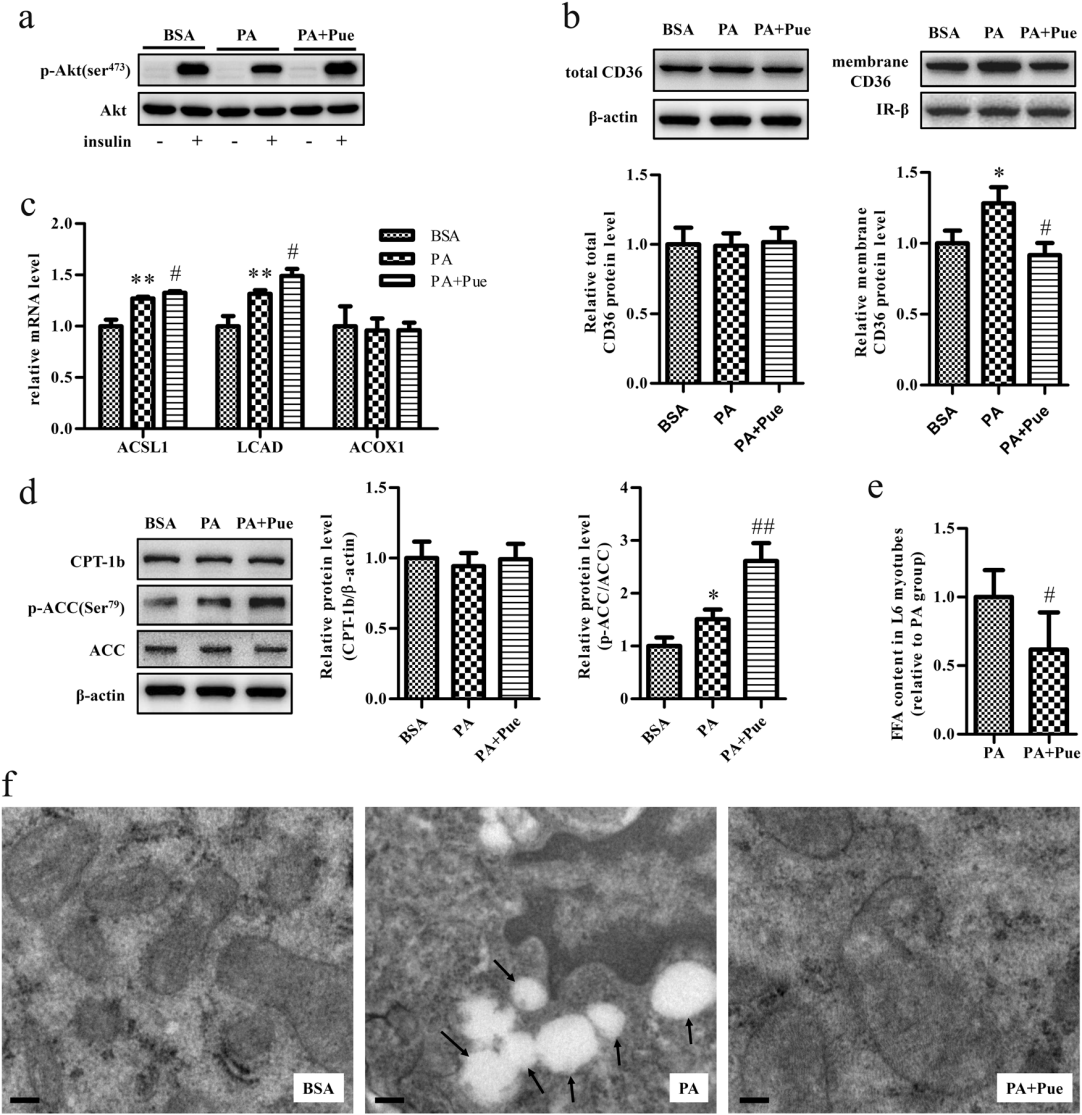
Puerarin promoted the oxidation of fatty acids in palmitate-induced insulin-resistant myotubes **a** Insulin-stimulated phosphorylation of Akt (Ser473) determined by western blot. Cells were incubated with 100 nM insulin during the last 20 min of treatment. **b** Western blot analyses and quantification of total and membrane CD36 in myotubes. IR-β was performed to confirm PM localization and normalization. **c** Relative mRNA expressions of ACSL1, LCAD, and ACOX1 analyzed by real-time RT-PCR and normalized to β-actin. **d** Western blot analyses and quantification of CPT-1b and p-ACC (Ser79)/ACC. **e** FFA content in cells normalized by respective protein content. **f** Representative electron micrographs showed the deposition of lipid droplets (arrowheads) in palmitate-treated myotubes but few lipids accumulation in cells pretreated with puerarin. Scale bar: 0.5 μm. All data were from three independent experiments and presented as mean ± s.d. *P < 0.05, **P < 0.01, vs. BSA group; ^#^*P* < 0.05, ^##^*P* < 0.01 vs. PA group. *PA* palmitate

To examine whether puerarin could directly act on muscle cells to promote the oxidation of fatty acids in vitro, L6 myotubes were cultured with 0.75 mM palmitate for 24 h to induce insulin resistance^16^. As was expected, insulin-stimulated phosphorylation of Akt (Ser473) was significantly decreased in L6 cells treated with palmitate in comparison with controls treated with vehicle (i.e., BSA). However, puerarin prevented this palmitate-induced insulin resistance (Fig. 5a).

In accordance with the findings of the in vivo study, puerarin prevented increases in membrane levels of CD36 in myotubes treated with palmitate (Fig. 5b). Interestingly, in response to the increased availability of palmitate, the level of phosphorylation of ACC, as well as the mRNA levels of acyl-CoA synthetase long-chain family member 1 (ACSL1) and LCAD, actually increased in insulin-resistant myotubes treated with palmitate (Fig. 5c and d), which implied an increase in the oxidation of fatty acids in these cells, which was in agreement with previous reports^16, 26, 27^. Nevertheless, pre-incubation with puerarin further increased the expression levels of the abovementioned genes regardless of the CPT-1b levels without significant differences between the three groups, which indicated that puerarin further increased the oxidation of fatty acids in myotubes treated with palmitate (Fig. 5c and d). Accordingly, puerarin significantly decreased the FFA content of muscle cells treated with palmitate (Fig. 5e). Likewise, TEM revealed the deposition of lipid droplets inside muscle cells treated with palmitate, whereas pretreatment with puerarin markedly reduced the accumulation of lipids (Fig. 5f). Taken together, these findings revealed that puerarin could directly act on muscle cells to promote the oxidation of fatty acids.

## Discussion

Skeletal muscle tissue requires an adequate supply of energy and is responsible for more than 80% of the insulin-stimulated metabolism of glucose in the body^28^. Hence, skeletal muscle plays an important role in the pathogenesis of insulin resistance and type 2 diabetes. Mitochondria are the principal energy-producing organelles and “burn” fuels via cellular respiration. Effective fuel switching in mitochondria requires sensitive signaling, which is presumably effectively coupled to mitochondrial function^29, 30^. Impairments in mitochondrial function that direct fatty acids toward storage instead of oxidation may contribute significantly to the accumulation of IMCLs, which directly causes and accelerates insulin resistance and type 2 diabetes^3, 4, 8^. In contrast to the liver, for example, the anabolic pathways in muscle, such as lipogenesis, glycerolipid synthesis, ketogenesis, and gluconeogenesis, are relatively inactive. These factors imply that mitochondria in muscle are highly sensitive to disturbances in fuel supply and as such may serve as indicators of energy metabolism disorders. Thus, a study of mitochondria in skeletal muscle in diabetic subjects will be informative. Here, the soleus muscle, which is a slow-twitch muscle, is rich in mitochondria, and sustains aerobic activity using fats and carbohydrates as fuels, was initially selected for testing.

Puerarin has wide-ranging effects on energy metabolism in the body. It exhibits hypoglycemic and hypolipidemic activity^14^ and prevents body weight gain in diabetic rodents. Puerarin was also found to increase the fecal excretion of TG, promote the oxidation of fatty acids, and suppress the synthesis of fats in the liver of HFD mice^12^. Furthermore, we revealed that puerarin facilitated the expression of mitochondrial UCP2 and UCP3 and increased the oxidation of lipids in muscle. These findings implied that puerarin could not only increase the consumption of energy in the body but also specifically facilitate the “burning” and disposal of lipids, which is a highly desirable function of puerarin that needed to be determined. Unfortunately, in previous studies, as well as in the current study, the energy consumption and respiratory quotient of animals treated with puerarin were not directly measured, which compromised our understanding of the effect of puerarin on systemic metabolism in vivo. Future studies should focus on this area.

Here, we focused on the effects of puerarin on mitochondrial function and the oxidation of fatty acids in muscle and found that puerarin improved mitochondrial performance, promoted the oxidation of fatty acids, and prevented the accumulation of IMCLs in the muscle of diabetic rats. Specifically, we detected increases in the expression levels of the main regulators of mitochondrial biogenesis and function, namely, SIRT1, PGC-1α, and its target TFAM^23, 24^, upon the administration of puerarin. More specifically, puerarin increased the copy number of mtDNA and the expression of the mitochondria-encoded gene MTCO1, as well as the density of mitochondria, in the muscle of diabetic rats. In addition, puerarin reversed mitochondrial dysfunction in skeletal muscle in diabetic rats by increasing the expression levels of genes involved in the TAC and OXPHOS^31–33^.

It is known that diabetes mellitus is closely associated with oxidative stress in tissue, including muscle^8^, which could cause mitochondrial abnormalities via damage to proteins, lipids, and DNA^5^. The redox enzyme p66Shc generates mitochondrial ROS via the oxidation of cytochrome c^34^. UCP2 and UCP3 can decrease the production of ROS via their proton-translocating activity and thus lower the mitochondrial membrane potential^35^. SOD2 is a major antioxidant enzyme in mitochondria. The mitochondrial deacetylase SIRT3 deacetylates lysine residues in SOD2 and thus promotes its antioxidant activity^36^. Puerarin protected mitochondria in muscle from oxidative damage by increasing the expression levels of SIRT3, SOD2, UCP2, and UCP3, as well as decreasing the phosphorylation of p66Shc, and thus enhanced theeffectiveness of antioxidant defense and reduced the production of ROS in diabetic rats, which was confirmed by TEM, which showed fewer adverse changes in the mitochondrial ultrastructure. Moreover, puerarin alleviated oxidative stress in the bloodstream, as evidenced by decreases in MDA levels and increases in total SOD activity. It is known that SOD3 is a more important antioxidant enzyme in blood^37^. However, in the current study, the method that was used only determined the total activity of SOD and could not distinguish between SOD3 and other forms of SOD. Future studies should address this issue. Nevertheless, our findings suggested that the beneficial effects of puerarin were not simply due to its antioxidant activity but rather occurred because it triggered a set of specific, yet unknown, biological processes^38, 39^. In addition, they indicated that skeletal muscle was not the only tissue where the effects of puerarin occurred.

The transport of fatty acids across the PM is a critical step involved in the regulation of lipid metabolism in muscle. CD36 has been shown to be involved in the uptake of fatty acids from the bloodstream into muscle cells^40^. A protein is trafficked between an endosomal pool and the PM in response to selected stimuli^41^. ACC, on the other hand, is a key regulator of the oxidation of fatty acids and catalyzes the conversion of acetyl-CoA to malonyl-CoA, which is a potent allosteric inhibitor of CPT-1b, to control the entry of fatty acyl-CoA into mitochondria^42^. AMPK inactivates ACC to facilitate the oxidation of fatty acids. In the present study, the administration of puerarin significantly decreased membrane levels of CD36 and increased the phosphorylation of AMPK and ACC to enhance the activity of CPT-1b, which thus reduced the uptake of fatty acids, promoted the transport of fatty acids into mitochondria for oxidation, and prevented the accumulation of IMCLs. This effect of puerarin acted, at least in part, directly on the muscle cells.

However, if the membrane levels of CD36 in muscle cells were lower and the uptake of lipids into the muscle was reduced by treatment with puerarin, how was one effect of puerarin achieved, namely, the decrease in blood TG and FFA levels? As discussed previously, puerarin could increase the fecal excretion of TG and influenced the activities of enzymes related to the hepatic metabolism of lipids, which promoted the oxidation of fatty acids and suppressed the synthesis of fats^12^. Thus, an increase in the blood clearance of TG and FFA in diabetic rodents might result from a reduction in the absorption of lipids and an increase in the consumption of lipids. Moreover, additional fatty acid transporters are known, for example, PM fatty acid-binding protein and fatty acid transport proteins. Regrettably, the involvement of these two types of transporter in the uptake of FFA into different organs induced by puerarin was not addressed in the current or previous studies and should be investigated in the future.

Previously, it has been shown that the glucose-reducing effect of puerarin in diabetic rodents is dependent on MOR^25^. We further revealed that MOR signaling improved the insulin sensitivity of skeletal muscle in diabetic rats treated with puerarin^17^. Here, we showed that MOR was probably involved in mitochondrial biogenesis induced by puerarin. However, the underlying mechanism was still largely unknown, although insulin sensitization facilitated by MOR might represent a mechanism by which puerarin improved mitochondrial function. It has been shown that insulin increased the phosphorylation of mitochondrial proteins involved in OXPHOS, the TAC, and the metabolism of fatty acids in human skeletal muscle in vivo^43^. The insulin sensitizer pioglitazone upregulated the expression of PGC-1α and restored mitochondrial function in insulin-resistant myotubes. Knockdown of insulin receptor by siRNA in C2C12 myotubes downregulated the expression of PGC-1α and impaired mitochondrial bioenergetics^44^. These findings support the hypothesis that insulin signaling regulates mitochondrial biogenesis and function in skeletal muscle.

In summary, we have provided new evidence that puerarin greatly improved the function of mitochondria in the muscle of diabetic rats by upregulating the expression of a range of genes involved in mitochondrial biogenesis, OXPHOS, the detoxification of ROS, and the oxidation of fatty acids and consequently preventing the accumulation of IMCLs. Because mitochondrial dysfunction is a central event in metabolic disorders that affect the whole body, in particular in type 2 diabetes, treatment with puerarin appears to be an attractive therapeutic strategy.
